# Muscle cell membrane damage by very low serum sodium

**Published:** 2009-11-12

**Authors:** Joye Varghese, Vallath Balakrishnan, Shine Sadasivan, Prem Nair, Venkadeswaran Anantha Narayanan

**Affiliations:** 1 Department of Gastroenterology, Amrita Institute of Medical Sciences and Research Centre, Cochin, India.

**Keywords:** Hyponatremia, thiazide diuretics

## Abstract

A 63-year-old male was admitted with complaints of upper gastrointestinal symptoms with fatigue and myalgia. Investigations revealed severe hyponatremia with elevated creatine phosphokinase levels. Following further workup, it was diagnosed as a case of hyponatremia induced rhabdomyolysis. Because of prompt correction of hyponatremia, his renal function was preserved and myoglobinuria induced renal failure was avoided. The importance of early recognition of this potentially dangerous condition is emphasized.

## Patient and case report

A 63-year-old male was admitted with a history of heartburn, vomiting, tiredness and myalgia of one-day duration. There was no abdomen pain, distension or jaundice. There was no history of similar episodes previously. For the preceding 30 years, the patient had been consuming alcohol once a week (60ml of brandy), however there was no history of alcoholic binge prior to the current onset of symptoms. There was no history suggestive of seizure disorders. He was on antihypertensive medications (amlodepine and metopronolol) for 15 years. On physical examination, the patient was drowsy with mild to moderate dehydration. His vitals were normal. General examination did not reveal any clue. Abdomen was soft. There was no organomegaly or tenderness. Other systems were clinically normal. Baseline blood tests such as hemoglobin, white blood cell counts, platelets and renal functions were within normal limits. His liver function test showed three fold raise of SGOT / SGPT ratio, otherwise appeared normal ( Table 1 ).

Transabdominal ultrasonography showed fatty liver with mild prostomegaly and gastroscopy revealed large hiatus hernia. His serum sodium level was 101mmol/L (normal 136–145 mmol/L). As patient already was on anti-hypertensive medications, electrocardiogram and cardiac markers were done to exclude a cardiac event. The values of serum creatine phosphokinase (CPK), CPK-MB and troponin I were 5773 U/L (n = <170 U/L), 96.8 IU/L (n = <23 IU/L) and 0.008ng/ml (n = <0.012ng/ml) respectively. Urine for myoglobulin was negative. He was diagnosed as a case of rhabdomyolysis based on elevated serum CPK with normal CPK-MB/troponin I level. Meanwhile, 100ml of 3% hypertonic saline infusion was given at a rate of 10ml/hour to correct hyponatremia. His serum sodium and CPK value were 124 mmol/L and 157 U/L on 3 ^rd^ day respectively ( Table 2 ). Patient’s general condition improved well and was discharged on 5 ^th^ day.

## Discussion

Clinical judgment of hyponatremia is often difficult because of its nonspecific symptoms such as anorexia, vomiting, muscle weakness, headache, confusion, convulsion and coma. Hyponatremia can occur in various conditions such as salt wasting nephropathy, cerebral salt wasting syndrome and excess body fluid loss by medications (ex: acetazolamide, amphotericin, thiazide diuretics, amiodarone, angiotensin II receptor blockers, bromocriptine, carbamazepine). In an event of acute onset hyponatremia (<120 mmol/L), though treating the primary underlying disease is an important, immediate correction of sodium is mandatory to avoid hyponatremia related morbidity and mortality.

In our case, other causes of rhabdomyolysis such as trauma, seizures (particularly alcoholc withdrawal seizures), drugs, inflammatory myopathy and hereditary metabolic myopathies were excluded by detailed history, clinical and biochemical examinations. Hyponatremia induced rhabdomyolysis is an uncommon phenomenon. It has been noticed among psychiatric patients [1, 2] who had had history of polydipsia. There are case reports stating that thiazide diuretics [3], proton pump inhibitor [4], sulfamethoxazole/trimethoprim [5], mannitol therapy for colonoscopy [6] and following PVP (photoselective vaporization of prostate) for benign prostate hypertrophy [7] had led to hyponatermia induced rhabdomyolysis. Though underlying pathophysiology for hyponatremia induced rhabdomyolysis is still obscure, the proposed mechanism is the malfunction of muscle cell membrane Na+ Ca+ pump that can lead to rise in intracellular Ca+ and activation of neural protease & lipase. These enzymes could be responsible for rhabdomyolysis [3, 8]. Interestingly, there are case reports stating that hypernatremia can also lead rhabdomyolysis by unknown mechanisms [9, 10]. Approximately 30% individuals with rhabdomyolysis develop acute renal failure because of low renal perfusion or direct toxic effect and/or cast formation of myoglobin [11, 12]. Delayed diagnosis and inadequate/over correction of hyponatremia may lead to numerous complications such as acute renal failure, central pontine myelinosis and even death.

In our case, the patient was admitted with gastrointestinal symptoms and was incidentally identified as hyponatremia associated rhabdomyolysis. He was treated promptly with 3% hypertonic saline. His serum sodium as well as CPK values returned to normal limit by 3 ^rd^ day and discharged on 5 ^th^ day. His renal function was normal during his hospital stay. Because of early correction of hyponatremia, there was no myoglobulinuria induced renal failure.

## Conclusion

This report highlights the importance of the entity known as “hyponatremia induced rhabdomyolysis” and also that prompt correction of hyponatremia would prevent rhabdomyolysis induced renal failure.

## Figures and Tables

**Table 1:** Results of liver function tests

WBC (ku/ml)	Platelet (ku/mL)	Urea (mg%)	Creatinine (mg%)	Total bilirubin (mg/dL)	Direct bilirubin (mg/dL)	AST (IU/L)	ALT (IU/L)	SAP (IU/L)	Total protein (gm/dL)	Serum albumin (gm/dL)	Serum globulin (gm/dL)
9.56	210	27.5	0.94	1.8	0.1	375.2	98.7	75.6	8.9	5.3	3.6

Hb, hemoglobin; WBC, white blood cell; AST, aspartate aminotransferase; ALT, alanine aminotransferase; SAP, serum alkaline phosphatase

**Table 2:** Serum sodium, potassium and creatine phosphokinase from day 1 to day 5

	Serum Na (mmol/L)	Serum K (mmol/L)	CPK (U/L)
Day 1	101.2	3.7	5773
Day 2	109.2	3.9	-
Day 3	124.2	3.8	157
Day 4	126.5	3.6	-
Day 5	129.2	3.7	76
